# Gender differences in internet gaming among university students: a discriminant analysis

**DOI:** 10.3389/fpsyg.2024.1412739

**Published:** 2024-11-06

**Authors:** Júlia Gisbert-Pérez, Manuel Martí-Vilar, Cesar Merino-Soto, Guillermo M. Chans, Laura Badenes-Ribera

**Affiliations:** ^1^Facultad de Psicología y Logopedia, Universitat de València, Valencia, Spain; ^2^Tecnológico de Monterrey, Institute for the Future of Education, Monterrey, Mexico; ^3^Instituto de Investigación de Psicología, Universidad de San Martín de Porres, Lima, Peru; ^4^Tecnológico de Monterrey, School of Engineering and Sciences, Mexico City, Mexico

**Keywords:** gaming, gender differences, gaming motivations, toxicity, higher education, educational innovation

## Abstract

**Introduction:**

Research on gaming and gaming habits has predominantly focused on younger populations, particularly males. The main objective of this study was to analyze gender-based differences in gamer profiles, considering variables related to gaming habits and the gaming community.

**Methods:**

A total of 180 Spanish university students currently engaged in video gaming (*M* = 21.51 years, *SD* = 3.09, 57.4% male) participated in the study by completing an online questionnaire addressing gaming characteristics such as the age of onset, gaming hours, motives of gaming, and perceived toxicity in the video gaming community.

**Results:**

The results revealed statistically significant gender differences in the age of gaming initiation, weekly gaming hours, community toxicity, and several gaming motivations, including customization, cognitive challenge, violent gratification, and social interaction. Linear discriminant analysis identified that higher scores in the age of initiation and customization, along with lower scores in violent gratification and community toxicity, formed the combination of predictor variables that most strongly distinguished between genders.

**Conclusion:**

Understanding these gender differences is essential for capturing current gaming trends and addressing the needs of diverse gamers. Finally, the potential clinical implications of these findings are discussed.

## Introduction

Historically, video gaming and males have been closely associated. Video games are interactive digital experiences that immerse players in virtual environments through gameplay mechanics, often centered around challenges, goals, and competition (Jenkins, 2009). These games are available on various platforms, including consoles, computers, and mobile devices, and span a wide range of genres, from action and adventure to simulation and role-playing. Despite this diversity, video gaming has traditionally been male-dominated. Professional esports players are overwhelmingly male (Paaßen et al., 2017), and the video game industry comprises mainly male developers (Kuss and Griffiths, 2012). In the early days of gaming, men created many games for a predominantly male audience (Lopez-Fernandez et al., 2019), which fostered a male-centric gaming culture. This culture has often overshadowed the presence of female gamers, even though women now represent 45% of gamers in Western markets (Entertainment Software Association, 2018).

Despite significant progress in promoting inclusivity and diversity within video games, persistent stereotypes and gender-related perceptions continue to affect the gaming community. Female gamers are often influenced by gaming-related stigmas that shape their self-perception. Some video game content still features the excessive sexualization and objectification of female characters, which can contribute to a hostile environment for women (Lopez-Fernandez et al., 2019; Lynch et al., 2016). As a result, female gamers frequently experience sexism, harassment, gender-based violence, and objectification (Belskie et al., 2023; Kuss et al., 2022). This stereotypical culture impedes the recognition of women as legitimate and skilled gamers (Vermeulen et al., 2017), limiting their ability to identify fully with the gaming community (Vermeulen et al., 2017; Lopez-Fernandez et al., 2019). Consequently, many women struggle to see themselves as gamers. Only 35% of women identify as gamers, compared to 51% of men (Le Ngoc, 2022).

Given video games’ male-centric design and the persistence of gender stigma, gender-related differences in gaming patterns are to be expected. This situation raises important questions about how gender influences game selection, the gaming experience, and the potential psychological effects of these differences.

Several studies have investigated the variables related to gaming preferences among male and female gamers. Research consistently shows that males tend to exhibit higher levels of engagement, resulting in differences in gaming frequency between genders. For example, Miezah et al. (2020) reported that males averaged 14.18 h of gaming per week, compared to 8.88 h for females. Similarly, van Rooij et al. (2014) found that males spent 15 h per week playing online video games, while females averaged 7 h. Homer et al. (2012) reported even more significant disparities, with males gaming for 43 h per week compared to 30 h for females and daily gaming durations of 6 versus 4 h, respectively. Although higher video game consumption is often linked to earlier initiation of gaming (Nakayama et al., 2020), studies specifically examining gender-based differences in this area remain limited.

Similarly, prevalence rates of Internet gaming disorder (IGD) differ by gender (Kuss et al., 2022). Stevens et al. (2021) conducted a meta-analysis of 53 studies published between 2009 and 2019. Their analysis revealed significant gender differences, with a male-to-female ratio 2.5:1 across 31 studies. Only 3 of these 31 studies (9.6%) reported a higher prevalence of IGD among females.

Research on gender preferences in video game genres (Wohn et al., 2020) has shown that female gamers participate in various genres (Lopez-Fernandez et al., 2019; Hassan et al., 2019; Lewis and Griffiths, 2011). However, the findings are mixed, with some studies suggesting that women tend to gravitate toward less aggressive or competitive games than men (Lange et al., 2021; López-Fernández et al., 2020; Phan et al., 2012). This discrepancy may stem from the addictive nature of competitive games and the sometimes toxic behavior within their communities. In competitive gaming, the use of verbal communication can expose players, especially women, to harassment and toxicity (Belskie et al., 2023; Bopp et al., 2022). As a result, many women choose not to identify as gamers, conceal their gender while playing, or avoid social and online games altogether, potentially missing out on the social and psychological benefits that gaming can offer (Kuss et al., 2022).

Players’ motivations for gaming differ by gender, as highlighted in a systematic review by Martucci et al. (2023). The review found that male players are more likely to engage in gaming for competitive reasons, while female players tend to use games for social and relational purposes. Interestingly, these motivations also vary by region. In Europe, women are more inclined to play for competition and to showcase their skills (Lewis and Griffiths, 2011; Laconi et al., 2017), whereas men often turn to gaming as a coping mechanism or to achieve success. In contrast, studies involving Spanish participants suggest that women’s primary motivation is to have fun (Labrador et al., 2022). In Asia, research indicates that women are more drawn to gaming for social interactions, while men play to pass the time. Meanwhile, in the United States, women are more likely to game for achievement, empowerment, fun, or to strengthen social relationships (Lopez-Fernandez et al., 2019).

In conclusion, few studies have thoroughly analyzed female gaming behavior, leading to mixed findings (Lopez-Fernandez et al., 2019; Kuss et al., 2022; Vermeulen et al., 2017; Lopez-Fernandez et al., 2019; Labrador et al., 2022; McLean and Griffiths, 2019). Given the disparities observed in recent research, a more comprehensive investigation into gender differences among gamers is essential.

The discrepancies identified in previous research may result from various methodological factors, such as using different instruments to measure study variables, sometimes without sufficient validation of their reliability and accuracy (Hassan et al., 2019; Lange et al., 2021; Labrador et al., 2022). Additionally, many studies have included samples with a broad age range, from preadolescents to adults or the general population, which can introduce further variation (Lange et al., 2021; Labrador et al., 2022). To address these issues, our study adopts a two-pronged approach: (1) focusing on a population with specific age and characteristics and (2) utilizing an instrument with well-established reliability and internal consistency. This approach ensures that our findings are more accurate and reliable for the target population.

Understanding these gender differences is crucial for grasping current gaming trends, reducing stereotypes, and fostering healthier gaming communities. Additionally, this insight can inspire more diverse and inclusive game design ideas, enabling the creation of gaming experiences that cater to the preferences and needs of all players, regardless of gender. This study is particularly relevant at the etic and emic levels, as variables like gaming motivations have been explored across cultures (Bäcklund et al., 2022). Applying an etic/emic approach in gender studies within gaming allows for the identification of universal patterns while also acknowledging the specific gender-related experiences that shape players’ preferences and behaviors.

### Aims of this study

Given the mixed results regarding gaming preferences and motivations, the main objective of this study is to analyze the differences in gamer profiles based on gender, focusing on gaming habits and community-related variables. Additionally, the research seeks to determine whether trends observed in larger populations hold within a university demographic. Specifically, we propose the following hypotheses:

*Hypothesis 1 (H1)*: A higher proportion of males engage in video gaming compared to females. This hypothesis is based on the observed discrepancy in statistical data regarding the number of male and female gamers (Kuss and Griffiths, 2012) and the tendency for video games to be designed with predominantly male-oriented content (Lopez-Fernandez et al., 2019).

*Hypothesis 2 (H2)*: Significant differences exist between male and female players regarding age of gaming initiation, weekly gaming hours, and experiences of community toxicity. Due to the gender-biased content of video games (Lopez-Fernandez et al., 2019), disparities in gaming habits are expected among college students, similar to those found in younger populations (Miezah et al., 2020; Homer et al., 2012; Phan et al., 2012). Specifically, we anticipate the following:

- *Females will report a later age of gaming initiation*.
- *Males will report more hours of gaming per week*.
- *Males will report experiencing higher levels of toxicity within gaming communities*.

*Hypothesis 3 (H3)*: There are notable differences in the gaming motivations of male and female players. Given the gendered nature of video game content (Lopez-Fernandez et al., 2019), it is expected that motivations for gaming will vary by gender, although existing literature presents mixed findings (Lewis and Griffiths, 2011; Laconi et al., 2017; Labrador et al., 2022).

*Hypothesis 4 (H4)*: Gaming motives are critical factors in distinguishing male and female gamer profiles.

## Materials and methods

### Research design and procedure

A cross-sectional research design was employed using an online survey distributed through social media platforms. The study adhered to the ethical principles outlined in the 1964 Declaration of Helsinki and its subsequent amendments or comparable ethical standards. Approval was obtained from the Institutional Review Board of the University of Valencia (UV-INV_ETICA-3179584).

The target population for this study consisted of university students, specifically active video game players. The inclusion criteria required participants to be Spanish-speaking university students, at least 18 years old, and actively engaged in playing video games during the study.

### Participants

The convenience sample consisted of 180 Spanish students, primarily from higher education, actively engaged in video gaming. The participants had an average age of 21.52 years (*SD* = 3.08, range: 18–33). The sample had a male representation of 58.3% (see Table 1). There was no statistically significant age difference between genders [*t*(178) = 0.14, *p* = 0.893, Hedges’ *g* = 0.02, 95% CI: −0.28, 0.32]. On average, participants began playing video games at 9.06 years (*SD* = 3.42, range: 3–18), with a mean weekly gaming time of 4.08 h (*SD* = 1.81, range: 1–7).

**Table 1:** Descriptive statistics for variables under study.

	*M*	SD	Min.	Max.	Sk	Kr
Age	21.52	3.08	18	33	1.25	1.39
Age of gaming initiation	9.06	3.42	3	18	0.50	−0.45
Gaming hours per week	4.08	1.81	1	7	0.14	−1.13
Video game toxicity	2.86	1.35	1	5	−0.02	−1.25
Motives of gaming						
Immersion	14.66	3.47	4	20	−0.48	0.11
Customization	14.86	3.86	4	20	−0.54	−0.20
Violent Gratification	11.42	4.25	4	20	−0.17	−0.72
Coping	14.11	3.61	4	20	−0.47	0.01
Fun	18.24	2.20	4	20	−1.90	8.37
Cognitive challenge	14.16	3.58	4	20	−0.66	0.42
Competition	12.88	3.91	4	20	−0.31	−0.49
Social Interaction	14.17	4.11	4	20	−0.78	0.08

Regarding educational background, 2.2% (*n* = 4) had completed secondary education, 38.9% (*n* = 70) had finished high school, 12.2% (*n* = 22) had completed vocational training, 36.1% (*n* = 65) held a university degree, and 10.6% (*n* = 19) had completed a master’s or postgraduate program (Table 2).

**Table 2:** Descriptive statistics for sociodemographic characteristics.

	*N*	*%*
Educational background completed		
Secondary education	4	2.2
High school education	70	38.9
Vocational training	22	12.2
University degree	65	36.1
Master’s or postgraduate	19	10.6
Institution type		
Public	155	86.1
Private	12	6.7
Location study center		
Valencia	167	92.8
Castilla La Mancha	4	2.2
Andalusia	4	2.2
Others	1	0.6
Monthly family income		
<€1,000	30	26.1
€1,001–2,000	47	17.2
€2001–3,000	59	32.8
€3,001–4,000	31	17.2
>€4,000€	12	6.7

Most students, 86.1% (*n* = 155), reported enrolling in public institutions, whereas 6.7% (*n* = 12) attended private institutions. Most study centers were in the Valencian community, representing 92.8% (*n* = 167) of the sample. Smaller proportions were from Castilla La Mancha and Andalusia (2.2%, *n* = 4) and other autonomous communities (0.6%, *n* = 1).

In terms of monthly family income, 32.8% of participants reported an income between €2001 and €3,000, 26.1% had an income between €1,001 and €2000, 17.2% reported earning either less than €1,000 or between €3,001 and €4,000, and 6.7% had a monthly income exceeding €4,000.

### Variables and instruments

#### Sociodemographic and gaming information

Participants provided demographic information, including age, biological sex, income, and educational level. Additionally, gaming-related characteristics were assessed, such as the age of gaming initiation, weekly hours spent gaming, and the perceived level of toxicity within the gaming community.

#### The scale of motives for video game playing (e-MUV)

The e-MUV instrument (López Fernández et al., 2019) consists of 32 items, each rated on a five-point Likert scale ranging from 1 (strongly disagree) to 5 (strongly agree). These items assess eight dimensions of gaming motivations: immersion (e.g., “*I feel immersed in a fantasy/fictional world*”), customization (e.g., “*I enjoy designing things in-game*”), violent gratification (e.g., “*I enjoy violence in games; the more, the better*”), coping (e.g., “*I forget my worries when I play*”), fun (e.g., “*I enjoy gaming*”), cognitive challenge (e.g., “*The games challenge me mentally*”), competition (e.g., “*I like to win*”), and social interaction (e.g., “*I make new friends*”). Higher scores in each dimension indicate a stronger motivation for that particular gaming aspect.

In the current sample, the integrity of the e-MUV measurement model was preserved, with ULSMV *χ*^2^ = 471.866 (*p* < 0.0, df = 436), CFI = 0.996, RMSEA = 0.021 (90% CI = 0.00, 0.034), SRMR = 0.060. As reported by López Fernández et al. (2019), Cronbach’s alpha for the dimensions ranged from 0.79 (cognitive challenge) to 0.89 (social interaction). In this study, omega reliability (BCA bootstrap 95% CI, 1000 samples) confirmed internal consistency across all dimensions: immersion = 0.818 (95% CI = 0.757, 0.866), customization = 0.887 (95% CI = 0.884, 0.915), violent gratification = 0.898 (95% CI = 0.866, 0.921), coping = 0.859 (95% CI = 0.811, 0.891), fun = 0.901 (95% CI = 0.833, 0.952), cognitive challenge = 0.861 (95% CI = 0.818, 0.894), social interaction = 0.831 (95% CI = 0.779, 0.867), and cognitive challenge = 0.855 (95% CI = 0.809, 0.888).

### Procedures

#### Data collection

The online survey was conducted using LimeSurvey and distributed through various social and academic networks. Participants were thoroughly informed about the study’s objectives, and before beginning the survey, they provided electronic, written consent. Only those who consented were able to proceed with the questionnaire. Participation was entirely voluntary, anonymous, and without compensation.

#### Statistical analysis

Descriptive statistics were first used to summarize the study’s demographic characteristics and critical variables. For continuous variables, means, standard deviations, and minimum and maximum values were calculated, while categorical variables were reported as frequencies and percentages. Skewness and kurtosis statistics were also examined to assess the distribution shape of continuous variables, helping to determine whether they followed a normal distribution (Kline, 2022).

Group comparisons assume the instrument’s psychometric properties are equivalent to the compared groups. To ensure this equivalence, we conducted a preliminary analysis of the e-MUV scores for males and females (using sex as a covariate) by detecting differential item functioning (DIF). Given the small group sizes, a non-parametric DIF analysis based on contingency tables was used (Fidalgo et al., 2004). Two tests for polytomous variables were applied, using the observed score (i.e., the sum of item responses) as the matching variable: the Mantel test *χ*^2^ (*M*-*χ*^2^) (Mantel, 1963; Zwick et al., 1993) and Generalized Mantel–Haenszel *χ*^2^ (GMH-*χ*^2^) (Zwick et al., 1993). These tests effectively identify different types of DIF (Zwick et al., 1993; Elosua and Wells, 2013; Fidalgo and Bartram, 2010): uniform DIF (related to scalar invariance of thresholds or intercepts) and non-uniform DIF (related to metric invariance of factor loadings). *p*-values were corrected by multiple testing using the Bonferroni adjustment (Penfield, 2001), resulting in a significance threshold of 0.002 for the four items in each e-MUV dimension.

Subsequently, a chi-square (*χ*^2^) test was used to compare the distribution of males and females in the player sample. Independent samples *t*-tests were conducted to examine gender differences across the variables under study. Before performing these analyses, we ensured that all statistical assumptions were met. Hedges’ *g* was used to measure effect size, providing an unbiased estimate of the standardized mean difference. Effect sizes of 0.15, 0.36, and 0.65 were interpreted as small, moderate, and large, respectively (Lovakov and Agadullina, 2021).

Finally, linear discriminant analysis (LDA) was conducted using the forward stepwise selection method (Tabachnick and Fidell, 2014) to identify the gaming-related variables that most effectively differentiate between men and women. Only statistically significant variables associated with the participant’s gender in prior analyses were included as predictors in the LDA. The selection criterion for predictor variables was based on minimizing Wilks’ Lambda (tolerance <0.001, entry *F* = 3.84, and removal *F* = 2.71). This stepwise method progressively added predictor variables to the discriminant function, optimizing the distinction between groups by minimizing within-group variation and maximizing between-group variation. The discriminant function was then used to classify participants into one of the two groups.

Several tests were used to evaluate the discriminant function’s fit (Tabachnick and Fidell, 2014): (1) Wilks’ Lambda and the *χ*^2^ significance test were employed to assess whether the group centroid scores were equal; (2) the percentage of variance explained by the discriminant function in group differences was measured using Canonical *R*^2^; (3) the predictive accuracy was evaluated by examining the percentage of correct classifications made by the discriminant function; and (4) the quality, stability, and generalizability of the discriminant function were tested using the “leave-one-out classification” technique, a cross-validation method (data resampling method). In this technique, all but one sample observation is used to develop the discriminant function, which is then applied to predict the group membership of the omitted observation. This process is repeated for each observation in the dataset, ensuring that every observation is classified by a function derived from the remaining data.

The LDA was performed using SPSS statistical software version 28 for Windows, with a significance level of 0.05 (two-tailed) assumed for all interpretations. The R packages used included *lavaan* (Rosseel, 2012), *MeasInv* (Wells, 2022), and *MBESS* (Kelley, 2023).

## Results

### Descriptive statistics

Table 1 presents the descriptive statistics for the variables under study. Notably, the univariate skewness and kurtosis values for all analyzed variables fall within the conventional normality threshold (Kline, 2022).

In terms of the perceived toxicity level of the video game community most frequented by the sample, 22.8% (*n* = 41) rated it as low, 18.3% (*n* = 33) as medium-low, 20.6% (*n* = 37) as medium, 26.7% (*n* = 48) as medium-high, and 11.7% (*n* = 21) as high.

### Differential item functioning

During the preliminary evaluation of the psychometric equivalence of the e-MUV between males and females, *p*-values for the M-*χ*^2^ (uniform DIF) and GHM-*χ*^2^ (non-uniform DIF) tests exceeded 0.002. These results indicate that no item exhibited significant uniform or non-uniform DIF (see Supplementary Table S1).

### Differences by gender in variables under study

Table 3 illustrates the outcomes of the Student’s *t*-test examining gender differences in the study variables. Initially, a statistically significant difference was found in the distribution of males and females in the sample [*χ*^2^(1) = 5.00, *p* = 0.025], indicating that the proportion of males (58.3%) and females (41.7%) deviated from what would be expected by chance. A significant difference was also observed in the age of video game initiation (*p* < 0.001), with males starting at a younger age (*M* = 7.84, *SD* = 2.60) compared to females (*M* = 10.76, *SD* = 3.64). Additionally, there were statistically significant differences in weekly gaming hours (*p* = 0.009) and perceived toxicity in the video gaming community (*p* < 0.001), with males reporting more weekly gaming hours and experiencing higher levels of toxicity. No significant differences between males and females were found in monthly family income [*χ*^2^(4) = 1.25, *p* = 0.869].

**Table 3:** Differences by gender in variables under study.

	Males		Females						
	*M*	*SD*	*M*	*SD*	*t*	df	*p*	*g*	95% CI
Age	21.54	3.08	21.48	3.10	0.14	178	0.893	0.02	−0.28, 0.32
Age of gaming initiation	7.84	2.68	10.76	3.64	−5.91	128.76	<0.001	−0.94	−1.24, −0.62
Gaming hours per week	4.39	1.78	3.66	1.78	2.66	177	0.009	0.40	0.10, 0.70
Video game toxicity	3.27	1.24	2.29	1.29	5.10	178	<0.001	0.77	0.46, 1.07
Immersion	14.41	3.28	15.01	3.71	−1.15	178	0.251	−0.17	−0.47, 0.12
Customization	13.86	3.67	16.25	3.70	−4.31	178	<0.001	−0.65	−0.95, −0.35
Violent gratification	12.75	3.54	9.56	4.49	5.13	135.38	<0.001	0.80	0.50, 1.11
Coping	14.04	3.43	14.21	3.86	−0.32	178	0.749	−0.48	−0.34, 0.25
Fun	18.31	1.96	18.13	2.51	0.54	178	0.588	0.08	−0.21, 0.38
Cognitive challenge	14.90	3.04	13.13	4.02	3.20	131.23	0.002	0.50	0.20, 0.80
Competition	14.10	3.24	11.19	4.14	5.08	134.68	<0.001	0.80	0.49, 1.10
Social Interaction	14.77	3.72	13.32	4.94	2.29	140.17	0.023	0.36	0.06, 0.65

M, mean; SD, standard deviation; df, degree of freedom; *g*, Hedge’s *g*.

**Figure 1:**
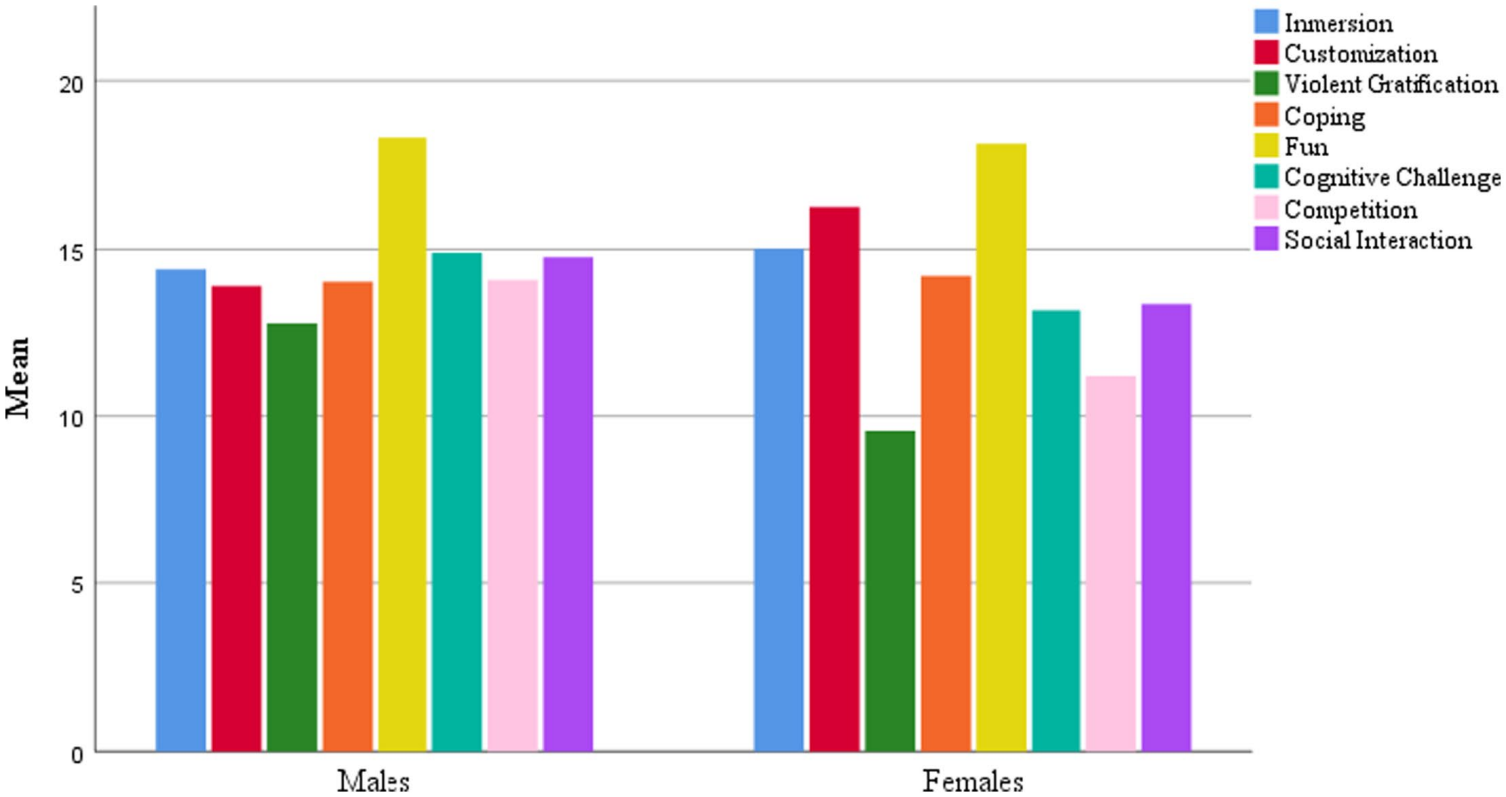
Mean distribution of gaming motivations (e-MUV) by gender.

Regarding the e-MUV dimensions, statistically significant differences were found in customization (*p* < 0.001), violent gratification (*p* < 0.001), cognitive challenge (*p* = 0.002), competition (*p* < 0.001), and social interaction (*p* = 0.023) (see Figure 1). Specifically, males showed higher motivations for violent gratification, cognitive challenge, competition, and social interaction, while females reported higher motivations for customization. No statistically significant differences were found between groups for the remaining variables.

### Linear discriminant analysis

A forward stepwise discriminant analysis was carried out, using the minimization of Wilks’ Lambda as the criterion for selecting predictor variables. Before the analysis, the statistical assumption was verified. The Box’s *M* test indicated a violation of the assumption of equality of covariance matrices (Box’s *M* = 36.70, *F* = 3.58, *p* < 0.001). However, this problem is not considered problematic (Tabachnick and Fidell, 2014) given the large sample size.

Since only two groups were analyzed, a single discriminant function was derived. The function demonstrated a statistically significant association between the groups and all predictor variables, accounting for 36.7% of the between-group variability. The overall Wilks’ lambda was statistically significant [Wilks’ lambda = 0.63, *χ*^2^(4) = 80.16, *p* < 0.001], indicating that the predictors effectively differentiated between the gender groups. Women had higher discriminant function scores (*M* = 0.69, *SD* = 0.46) compared to men (*M* = 0.12, *SD* = 0.33), and this difference was statistically significant [*t*(125.70) = −9.10, *p* = <0.001, Hedges’ *g* = −1.45, 95% IC: −1.78, −1.11].

Table 4 displays the within-group correlations among the predictors, the discriminant function, and the standardized weights. The correlation coefficients represent Pearson correlations between the predictors and the discriminant function. Notably, all coefficients exceeded 0.30, a commonly accepted threshold indicating the relevance of predictor variables in discriminant function analysis (Burns and Burns, 2008). The strongest relationships with the discriminant function were observed for the age of gaming initiation (*r* = 0.61) and the violent gratification dimension (*r* = −0.51), followed by community toxicity (*r* = −0.49) and customization (*r* = 0.41). Specifically, a later age of gaming initiation and higher customization, along with lower levels of violent gratification and community toxicity, were associated with higher discriminant function scores.

**Table 4:** Results of discriminant analysis.

Predictors	Structure coefficients	Standardized coefficients	Pooled within-group correlations among predictors		
			Video game toxicity	Customization	Violent gratification
Age of gaming initiation	0.61	0.67	0.14	−0.02	−0.05
Video game toxicity	−0.49	−0.47		−0.07	0.23
Customization	0.41	0.40			0.02
Violent gratification	−0.51	−0.38			
Canonical *R*	0.61				
Eigenvalue	0.58				

Table 5 shows the classification results using the “leave-one-out” cross-validation method (jackknifed classification). The overall accuracy rate was 80.0%, correctly classifying 80.0% of respondents into “male” or “female” groups. Males were classified with slightly higher accuracy (87.6%) than females (69.3%). These results were consistent in the cross-validated classification (Table 5).

**Table 5:** Classification results using the “leave-one-out” cross-validation method.

		Predicted group membership		% cases
Sample	Gender	Men	Women	Correctly classified
Original	Men	92	13	87.6
	Women	23	52	69.3
	% Total cases correctly classified			80.0
Cross-validated	Men	92	13	87.6
	Women	23	52	69.3
	% Total cases correctly classified			80.0

## Discussion

This study explores the differences between male and female gamer profiles, focusing on variables related to gaming habits, motivations, and characteristics such as community toxicity. The video game industry and its content reflect inherent gender disparities, possibly contributing to divergent gaming profiles and outcomes (Kuss et al., 2022). While certain factors like game genre preferences and gaming motivations, have been extensively studied, the findings remain inconsistent (Lopez-Fernandez et al., 2019; Lopez-Fernandez et al., 2019). Less examined variables, like gaming community toxicity, may have a distinct gendered influence and deserve further investigation (Belskie et al., 2023).

The results support hypothesis 1 (H1), revealing a higher proportion of male gamers in the sample (58.3%). These findings align with previous studies (Lopez-Fernandez et al., 2019; Phan et al., 2012), challenging the notion that female gamers constitute a minority (Entertainment Software Association, 2018). While many video games still target primarily male audiences (Lopez-Fernandez et al., 2019), these results may suggest that the industry is gradually shifting toward creating games that also appeal to women.

The results also support Hypothesis 2 (H2), highlighting differences between males and females in the age of gaming initiation, weekly gaming hours, and the perceived toxicity of video gaming communities. As expected, female gamers reported a later gaming onset than males (H2a). This delay may be attributed to the widespread belief that video games are primarily designed for a male audience (Lopez-Fernandez et al., 2019). Consequently, males reported more weekly gaming hours (H2b), consistent with Nakayama et al.’s findings (Nakayama et al., 2020), which link earlier gaming initiation to increased gaming activity. Additionally, male gamers were more likely to engage in toxic gaming communities than their female counterparts (H2c).

This trend may be related to the traditional association of males with competitive and violent video games (Lange et al., 2021; Bopp et al., 2022). Female gamers, on the other hand, may avoid toxic communities in competitive games due to the risk of encountering sexism or harassment, prioritizing their psychological well-being over gaming interests (Belskie et al., 2023; Kuss et al., 2022). For women, the decision to play certain video games is often influenced by the need for psychological safety rather than purely personal preferences, given the potential risks associated with toxic gaming environments (Belskie et al., 2023).

Our findings partially support Hypothesis 3 (H3), revealing significant differences in gaming motivations between male and female players. Distinctions were observed in customization, violent gratification, cognitive challenge, competition, and social interaction. Consistent with previous research (López-Fernández et al., 2020; Gursesli et al., 2024), male gamers exhibited higher motivation for competition, violent gratification, and social interaction, whereas female gamers were more motivated by customization and creative aspects of video games. This discrepancy may be due to female gamers’ tendency to avoid competitive, toxic, or socially interactive games, particularly those that require revealing their identity, such as through audio channels (Belskie et al., 2023). Additionally, male gamers demonstrated a greater motivation for cognitively challenging video games.

Male gamers’ motivations often align with genre preferences, such as shooters (Phan et al., 2012; Kim et al., 2016). Shooters and massively multiplayer online role-playing games (MMORPGs) are frequently associated with gaming disorder (Na et al., 2017), with social interaction motivation linked to increased time spent in online games (Király et al., 2017). Both social interaction and the pursuit of violent gratification have been implicated in the development of problematic gaming behaviors (Lopez-Fernandez et al., 2019). In contrast, female gamers’ preference for customization and creation is typically associated with construction and role-playing games. Notably, customization has been found to have a negative relationship with the development of problematic gaming (López-Fernández et al., 2020).

The findings supporting H2 and H3 are consistent with gender socialization theory (Bem, 1993) and social identity theory (Tajfel and Turner, 1979). Gender socialization theory (Bem, 1993) suggests that from an early age, boys and girls socialize differently regarding their interests and activities. Due to cultural and social stereotypes, video games are often more closely associated with male interests. This situation may explain why females start playing video games later and spend fewer hours per week gaming. Social identity theory (Tajfel and Turner, 1979) also posits that individuals tend to identify with groups that reinforce their sense of identity. Men may be more attracted to specific video games because they provide an opportunity to strengthen their masculine identity, particularly in genres that emphasize competition and action. This tendency to seek out competitive and action-oriented games could also explain the greater participation of men in toxic gaming communities, where competition and aggression are more prevalent.

Building on these findings, Hypothesis 4 (H4) is supported. The study suggests that distinct gender patterns emerge based on gaming motivations, the age of gaming initiation, and the perceived toxicity of video gaming communities. Through linear discriminant analysis, the variables most strongly associated with the discriminant function were the age of initiation and the gaming motive of violent gratification, followed by community toxicity and the gaming motive of customization. Higher scores for age of initiation (indicating older ages) and customization, combined with lower scores for violent gratification and toxicity, represent the most practical combination of predictor variables for distinguishing between genders.

Several conclusions can be drawn from these findings. First, there is an apparent gender disparity in the age of video game initiation, with male gamers being exposed to the positive and negative effects of video gaming at an earlier age. Second, gaming motivations differ by gender, suggesting that male and female gamers have distinct reasons for engaging in video games. Consequently, it can be inferred that they may not gravitate toward the same types of games (Lange et al., 2021; López-Fernández et al., 2020; Bopp et al., 2022). Third, this difference in video gaming preferences leads to varied exposure to toxic communities. Women may avoid certain video games that involve female objectification or communities where they are likely to encounter sexism or harassment due to high toxicity levels (Belskie et al., 2023). While this avoidance highlights the persistence of gender stereotypes in video gaming, it can also have positive outcomes. It promotes healthier video gaming habits among female players, characterized by a later onset of gaming, more creative motivations, and participation in less toxic communities.

Finally, the predictor variables associated with male gamers, such as higher scores in violent gratification and community toxicity, are positively linked to the development of gaming disorder. In contrast, the predictor variables associated with female gamers show the opposite effect (Lopez-Fernandez et al., 2019; Kim et al., 2016).

### Limitations and future avenues of research

Several limitations of the current study should be acknowledged. First, there may be selection bias in the sample, as the accessibility of the questionnaires may have favored certain groups, such as those with access to computers. The sample, composed predominantly of university students, also limits the generalizability of the findings to broader age populations. Additionally, the study relies solely on observed variables, introducing some measurement error by not incorporating unobservable variables that could be estimated through Structural Equation Modeling (SEM) or Item Response Theory (IRT). Using latent variables would help control for variance unrelated to the construct.

Furthermore, future research could benefit from a more extensive and diverse sample, including gamers of various ages, to examine the stability of gender-related predictor variables across different age groups. Exploring the consistency of results in multicultural samples would also be valuable. Lastly, the development and validation of questionnaires specifically designed to assess constructs related to video games, such as toxicity, is recommended.

### Practical implications

Understanding the disparities between male and female gamers is crucial for gaining insights into current gaming trends. The findings, which highlight gender differences, stress the importance of reducing gender stereotypes and fostering healthier gaming communities to promote more equitable gaming experiences. This perspective also underscores the need for more inclusive game design, offering gaming experiences that align with the preferences and needs of players across genders.

The study has clinical implications as well, suggesting that gender-specific profiles may be linked to problematic gaming. This notion highlights the importance of developing tailored prevention programs that address the unique challenges faced by individuals with diverse gender identities in the context of gaming-related disorders. Such targeted interventions could lead to more effective, personalized strategies for mitigating the risks of excessive gaming behaviors.

Addressing the toxicity in gaming communities is also essential, as it can have severe psychological effects. Specific actions must be taken from both educational and familial perspectives. Schools and communities should implement educational programs that promote gender equity in gaming spaces, including workshops to teach young people about the importance of diversity, inclusion, and respect in gaming, with family involvement. While video game design is becoming more inclusive, the gaming community must also embrace these initiatives. Cultivating a culture of respect and equity early on is crucial for creating lasting behavioral change within gaming communities.

## Conclusion

This study provides new insights into the impact of gender on gaming behaviors. The results highlight gender differences in the age of gaming initiation, gaming hours, motivations for gaming, and the toxicity levels within gaming communities. Additionally, the identified predictor variables—age of gaming onset, gaming motivations (customization and violent gratification), and community toxicity—further underscore the distinctions between male and female gamers. These findings emphasize the importance of examining the gaming population, focusing on gender differences and the underlying factors that contribute to them.

In conclusion, fostering more inclusive game design and taking concrete steps in education and community involvement can help create a healthier and more equitable gaming environment. Addressing these issues is essential for players’ mental well-being and cultivating a more positive and enriching gaming culture.

## Data Availability

The raw data supporting the conclusions of this article will be made available by the authors, without undue reservation.

## References

[ref1] Bäcklund C. Elbe P. Gavelin H. M. Sörman D. E. Ljungberg J. K. (2022). Gaming motivations and gaming disorder symptoms: a systematic review and meta-analysis. J. Behav. Addict. 11, 667–688. doi: 10.1556/2006.2022.00053, PMID: 36094861 PMC9872536

[ref2] Belskie M. Zhang H. Hemminger B. M. (2023). Measuring toxicity toward women in game-based communities. J. Electron. Gaming Esports 1:35. doi: 10.1123/jege.2022-0035

[ref3] Bem S. L. (1993). The lenses of gender: transforming the debate on sexual inequality. New Haven: Yale University Press.

[ref4] Bopp T. Karadakis K. Shay M. (2022). “Uh oh, we have an Egirl”: when, where, and how gender influences gaming. JPESM. 9, 12–21. doi: 10.15640/jpesm.v9a2

[ref5] Burns R. P. Burns R. (2008). Business research methods and statistics using SPSS. London: Sage.

[ref6] Elosua P. Wells C. (2013). Detecting DIF in polytomous items using MACS, IRT and ordinal logistic regression. Psicológica 34, 327–342.

[ref7] Entertainment Software Association. Essential facts about the computer and video game industry. (2018). Available at: https://www.theesa.com/resource/2018-essential-facts-about-the-computer-and-video-game-industry/

[ref8] Fidalgo Á. M. Bartram D. (2010). A comparison between some generalized Mantel-Haenszel statistics for detecting DIF in data simulated under the graded response model. Appl. Psychol. Meas. 34, 600–606. doi: 10.1177/0146621610378405

[ref9] Fidalgo Á. M. Ferreres D. Muñiz J. (2004). Utility of the Mantel-Haenszel procedure for detecting differential item functioning in small samples. Educ. Psychol. Meas. 64, 925–936. doi: 10.1177/0013164404267288

[ref10] Gursesli M. C. Martucci A. Mattiassi A. D. A. Duradoni M. Guazzini A. (2024). Development and validation of the psychological motivations for playing video games scale (PMPVGs). Simul. Gaming 55, 856–885. doi: 10.1177/10468781241260861

[ref11] Hassan H. Mailok R. Hashim M. (2019). Gender and game genres differences in playing online games. J. ICT Educ. 6, 1–15. doi: 10.37134/jictie.vol6.1.2019

[ref12] Homer B. D. Hayward E. O. Frye J. Plass J. L. (2012). Gender and player characteristics in video game play of preadolescents. Comput Human Behav. 28, 1782–1789. doi: 10.1016/j.chb.2012.04.018

[ref13] Jenkins H. (2009). “Game design as narrative architecture” in First person: new media as story, performance, and game. eds. Wardrip-Fruin N. Harrigan P. (Cambridge, MA: MIT Press), 118–130.

[ref14] Kelley K. (2023). MBESS: the MBESS R package. R package (version 4.9.3). Available at: https://CRAN.R-project.org/package=MBESS

[ref15] Kim N. R. Hwang S. S.-H. Choi J.-S. Kim D.-J. Demetrovics Z. Király O.. (2016). Characteristics and psychiatric symptoms of internet gaming disorder among adults using self-reported DSM-5 criteria. Psychiatry Investig. 13, 58–66. doi: 10.4306/pi.2016.13.1.58, PMID: 26766947 PMC4701686

[ref16] Király O. Tóth D. Urbán R. Demetrovics Z. Maraz A. (2017). Intense video gaming is not essentially problematic. Psychol. Addict. Behav. 31, 807–817. doi: 10.1037/adb0000316, PMID: 28956935

[ref17] Kline R. B. (2022). “Principles and practice of structural equation modeling” in. eds. Kenny D. A. Little T. D.. 3rd ed (New York, NY: Guilford Press).

[ref18] Kuss D. J. Griffiths M. D. (2012). Internet gaming addiction: a systematic review of empirical research. Int J Ment Health Addict. 10, 278–296. doi: 10.1007/s11469-011-9318-5

[ref19] Kuss D. J. Kristensen A. M. Williams A. J. Lopez-Fernandez O. (2022). To be or not to be a female gamer: a qualitative exploration of female gamer identity. Int. J. Environ. Res. Public Health 19:1169. doi: 10.3390/ijerph19031169 35162194 PMC8835226

[ref20] Labrador F. J. Fernández-Arias I. Martín-Ruipére S. Bernaldo-de-Quirós M. Vallejo-Achón M. Sánchez-Iglesias I.. (2022). Women and videogames: what do they play? An Psicol. 38, 508–517. doi: 10.6018/analesps.504281

[ref21] Laconi S. Pirès S. Chabrol H. (2017). Internet gaming disorder, motives, game genres and psychopathology. Comput Human Behav. 75, 652–659. doi: 10.1016/j.chb.2017.06.012

[ref22] Lange B. P. Wühr P. Schwarz S. (2021). Of time gals and mega men: empirical findings on gender differences in digital game genre preferences and the accuracy of respective gender stereotypes. Front. Psychol. 12:12. doi: 10.3389/fpsyg.2021.657430, PMID: 34040565 PMC8141853

[ref23] Le Ngoc M. T.. Shining the spotlight on female gamers. (2022). Available at: https://newzoo.com/resources/blog/shining-the-spotlight-on-female-gamers

[ref24] Lewis A. Griffiths M. (2011). Confronting gender representation: a qualitative study of the experiences and motivations of female casual-gamers. Aloma Rev. Psicol. Ciències l'Educació l'Esport. 28, 245–273.

[ref25] López Fernández F. Ortet-Walker J. Gallego S. Ortet-Fabregat G. (2019). Preliminary psychometric study of the e-MUV video game use motive scale. Àgora de Salut. 6, 181–188. doi: 10.6035/AgoraSalut.2019.6.19

[ref26] López-Fernández F. J. Mezquita L. Griffiths M. D. Ortet G. Ibáñez M. I. (2020). The role of personality on disordered gaming and game genre preferences in adolescence: gender differences and person-environment transactions. Adicciones 33, 263–272. doi: 10.20882/adicciones.1370 32100046

[ref27] Lopez-Fernandez O. Williams A. J. Griffiths M. D. Kuss D. J. (2019). Female gaming, gaming addiction, and the role of women within gaming culture: a narrative literature review. Front Psychiatry 10:10. doi: 10.3389/fpsyt.2019.00454, PMID: 31354536 PMC6635696

[ref28] Lopez-Fernandez O. Williams A. J. Kuss D. J. (2019). Measuring female gaming: gamer profile, predictors, prevalence, and characteristics from psychological and gender perspectives. Front. Psychol. 10:898. doi: 10.3389/fpsyg.2019.00898, PMID: 31105622 PMC6498967

[ref29] Lovakov A. Agadullina E. (2021). Empirically derived guidelines for effect size interpretation in social psychology. Eur. J. Soc. Psychol. 51, 485–504. doi: 10.1002/ejsp.2752

[ref30] Lynch T. Tompkins J. E. van Driel I. I. Fritz N. (2016). Sexy, strong, and secondary: a content analysis of female characters in video games across 31 years. J. Commun. 66, 564–584. doi: 10.1111/jcom.12237

[ref31] Mantel N. (1963). Chi-Square tests with one degree of freedom; extensions of the Mantel-Haenszel procedure. J. Am. Stat. Assoc. 58, 690–700. doi: 10.2307/2282717

[ref32] Martucci A. Gursesli M. C. Duradoni M. Guazzini A. (2023). Overviewing gaming motivation and its associated psychological and sociodemographic variables: a PRISMA systematic review. Hum Behav Emerg. 2023, 1–156. doi: 10.1155/2023/5640258

[ref33] McLean L. Griffiths M. D. (2019). Female gamers’ experience of online harassment and social support in online gaming: a qualitative study. Int J Ment Health Addict. 17, 970–994. doi: 10.1007/s11469-018-9962-0

[ref34] Miezah D. Batchelor J. Megreya A. M. Richard Y. Moustafa A. A. (2020). Video/computer game addiction among university students in Ghana: prevalence, correlates and effects of some demographic factors. Psychiatr Clin Psychopharmacol. 30, 1–23. doi: 10.5455/PCP.20200320092210

[ref35] Na E. Choi I. Lee T.-H. Lee H. Rho M. J. Cho H.. (2017). The influence of game genre on internet gaming disorder. J. Behav. Addict. 6, 1–8. doi: 10.1556/2006.6.2017.033, PMID: 28658960 PMC5520129

[ref36] Nakayama H. Matsuzaki T. Mihara S. Kitayuguchi T. Higuchi S. (2020). Relationship between problematic gaming and age at the onset of habitual gaming. Pediatr. Int. 62, 1275–1281. doi: 10.1111/ped.14290, PMID: 32379947

[ref37] Paaßen B. Morgenroth T. Stratemeyer M. (2017). What is a true gamer? The male gamer stereotype and the marginalization of women in video game culture. Sex Roles 76, 421–435. doi: 10.1007/s11199-016-0678-y

[ref38] Penfield R. D. (2001). Assessing differential item functioning among multiple groups: a comparison of three Mantel-Haenszel procedures. Appl. Meas. Educ. 14, 235–259. doi: 10.1207/S15324818AME1403_3

[ref39] Phan M. H. Jardina J. R. Hoyle S. Chaparro B. S. (2012). Examining the role of gender in video game usage, preference, and behavior. Proc. Human Factors Ergonomic. Soc. Annual Meeting 56, 1496–1500. doi: 10.1177/1071181312561297

[ref40] Rosseel Y. (2012). Lavaan: an R package for structural equation modeling. J. Stat. Softw. 48, 1–36. doi: 10.18637/jss.v048.i02

[ref41] Stevens M. W. Dorstyn D. Delfabbro P. H. King D. L. (2021). Global prevalence of gaming disorder: a systematic review and meta-analysis. Aust. N. Z. J. Psychiatry 55, 553–568. doi: 10.1177/0004867420962851, PMID: 33028074

[ref42] Tabachnick B. Fidell L. S. (2014). Using multivariate statistics. 6th Edn. Upper Saddle River, NJ: Pearson Education.

[ref43] Tajfel H. Turner J. C. (1979). “An integrative theory of intergroup conflict” in The social psychology of intergroup relations. eds. Austin W. G. Worchel S. (Monterey, CA: Brooks/Cole), 33–47.

[ref44] van Rooij A. J. Kuss D. J. Griffiths M. D. Shorter G. W. Schoenmakers T. M. van de Mheen D. (2014). The (co-)occurrence of problematic video gaming, substance use, and psychosocial problems in adolescents. J. Behav. Addict. 3, 157–165. doi: 10.1556/jba.3.2014.013, PMID: 25317339 PMC4189309

[ref45] Vermeulen L. Van Bauwel S. Van Looy J. (2017). Tracing female gamer identity. An empirical study into gender and stereotype threat perceptions. Comput Human Behav. 71, 90–98. doi: 10.1016/j.chb.2017.01.054

[ref46] Wells C. S.. (2022). MeasInv: collection of methods to detect dichotomous and polytomous differential item functioning (DIF). R package version 0.1.0. Available at: https://github.com/cswells1/MeasInv/

[ref47] Wohn D. Y. Ratan R. Cherchiglia L. (2020). “Gender and genre differences in multiplayer gaming motivations” in HCI in games (Cham: Springer International Publishing).

[ref48] Zwick R. Donoghue J. R. Grima A. (1993). Assessment of differential item functioning for performance tasks. J. Educ. Meas. 30, 233–251. doi: 10.1111/j.1745-3984.1993.tb00425.x

