# Supplementary material for: Gender differences in internet gaming among university students: a discriminant analysis
Source: Front Psychol. 2024 Nov 6;15:1412739. doi: 10.3389/fpsyg.2024.1412739 (PMC11577638; doi:10.3389/fpsyg.2024.1412739)
Supplement: Supplementary file 1 [file Data_Sheet_1.pdf]

Table S1

DIF analysis for e-MUV items

e-MUV Dimensiones	M-χ^2	*p*-value	GMH-χ^2	*p*-value
Immersion				
Muv1	3.437	0.064	– [a]	–
Muv2	0.003	0.956	–	–
Muv3	2.837	0.092	–	–
Muv4	0.066	0.797	–	–
Customization				
Muv5	0.077	0.781	3.013	0.556
Muv6	0.021	0.885	2.393	0.664
Muv7	1.004	0.316	4.506	0.342
Muv8	0.335	0.563	3.494	0.479
Violent Gratification				
Muv9	2.928	0.087	3.463	0.484
Muv10	2.648	0.104	4.793	0.309
Muv11	0.159	0.690	1.744	0.783
Muv12	8.161	0.004	8.354	0.079
Coping				
Muv13	0.015	0.903	0.694	0.952
Muv14	8.511	0.004	13.670	0.008
Muv15	2.849	0.091	9.559	0.049
Muv16	1.291	0.256	4.423	0.352
Fun				
Muv17	0.022	0.882	–	–
Muv18	1.296	0.255	–	–
Muv19	0.141	0.707	–	–

Muv20	0.277	0.599	–	–
Cognitive Challenge				
Muv21	0.125	0.724	1.072	0.899
Muv22	0.229	0.632	4.955	0.292
Muv23	3.211	0.073	4.948	0.293
Muv24	4.128	0.042	2.233	0.693
Competition				
Muv26	0.229	0.632	–	–
Muv27	3.211	0.073	–	–
Muv28	4.128	0.042	–	–
Social Interaction				
Muv29	2.693	0.101	3.648	0.456
Muv30	0.747	0.387	1.541	0.819
Muv31	0.228	0.633	2.751	0.600
Muv32	0.361	0.548	1.762	0.779

Note. M-χ^2: Mantel test. GMH-χ^2: generalized Mantel-Hanszel test. [a] Estimates not obtained due to insufficient data in one of the score strata.
